# Analytic Eigenbranches in the Semi-classical Limit

**DOI:** 10.1007/s11785-020-01011-4

**Published:** 2020-06-17

**Authors:** Stefan Haller

**Affiliations:** Department of Mathematics, University of Vienna, Oskar-Morgenstern-Platz 1, 1090 Vienna, Austria

**Keywords:** Semi-classical limit, Analytic eigenbranches, Witten Laplacian, 35P20

## Abstract

We consider a one parameter family of Laplacians on a closed manifold and study the semi-classical limit of its analytically parametrized eigenvalues. Our results establish a vector valued analogue of a theorem for scalar Schrödinger operators on Euclidean space by Luc Hillairet which applies to geometric operators like Witten’s Laplacian on differential forms.

Let *M* be a closed smooth manifold and suppose *E* is a complex vector bundle over *M*. Fix a smooth volume density on *M* and a smooth fiber wise Hermitian metric on *E*. We will denote the associated $L^2$ scalar product on the space of sections, $\Gamma (E)$, by $\langle \!\langle -,-\rangle \!\rangle$ and write $\Vert {-}\Vert$ for the corresponding $L_2$ norm.

Consider a one parameter family of operators acting on $\Gamma (E)$,

1 $$\begin{aligned} \Delta _t=\Delta +tA+t^2V,\qquad t\in \mathbb R, \end{aligned}$$

where $\Delta$ is a selfadjoint (bounded from below) Laplacian and $A,V\in \Gamma ({{\,\mathrm{end}\,}}(E))$ are smooth symmetric sections. This is a selfadjoint holomorphic family of type (A) in the sense of [9, Section VII§2]. According to the Kato–Rellich theorem, see [9, Theorem VII.3.9], its eigenvalues can be organized in analytic families referred to as *analytic eigenbranches* of $\Delta _t$. More precisely, there exist eigenbranches $\lambda _t$ and eigensections $\psi _t$, both analytic in $t\in \mathbb R$, such that

2 $$\begin{aligned} \Delta _t\psi _t=\lambda _t\psi _t\qquad \text {and}\qquad \Vert \psi _t\Vert =1. \end{aligned}$$

Furthermore, it is possible to choose a sequence of analytic eigenbranches, $\lambda ^{(k)}_t$, and corresponding analytic eigensections, $\psi _t^{(k)}$, such that at every time *t*, the sequence $\lambda _t^{(k)}$ exhausts all of the spectrum of $\Delta _t$, including multiplicities, and $\psi _t^{(k)}$ forms a complete orthonormal basis of eigensections. The analytic parametrization of the spectrum, $\lambda ^{(k)}_t$, is unique up to renumbering. The eigensections, on the other hand, are by no means canonical, and it seems more natural to consider the spectral projections instead.

In this note we study the semi-classical limit of the analytic eigenbranches, i.e., the behavior of $\lambda _t$ as $t\rightarrow \infty$. We will show that $t^{-2}\lambda _t$ converges to a finite limit $\mu$, see part (b) of the theorem below. Moreover, if the potential *V* is scalar valued, i.e., if $V=v\cdot {\text {id}}_E$ for a smooth function *v*, then $\mu$ has to be a critical value of *v*, see part (h) in the theorem below. These observations are analogous to a result of Luc Hillairet [8] who considered the scalar case on $M=\mathbb R^n$ with $A=0$.

While Hillairet’s proof uses the invariance of semi-classical measures under the corresponding Hamiltonian flow, our approach is entirely elementary and does not make use of these concepts. The ideas entering into the proof, however, appear to be essentially the same. Notably, in order to show that $\mu$ has to be a critical value of *V*, we too rely on commutator computations. Avoiding semi-classical measures makes the generalization to the vector valued case considered here straight forward. As in Hillairet’s argument, convergence of $t^{-2}\lambda _t$ follows from the fact that this quantity, suitably corrected due of the presence of *A*, is bounded and monotone, cf. (14) below. The fact that the Laplacian is semi-bounded enters crucially at this point.

Let us emphasize that analytically parametrized eigenbranches may cross and will in general not remain in the same order. Hence, the asymptotics of analytic eigenbranches might be quite different from the well understood asymptotics of the spectral distribution function, i.e., the asymptotics of the eigenvalues ordered increasingly. In particular, we cannot rule out the existence of analytic eigenbranches which do not correspond to any eigenvalue of the approximating harmonic oscillator associated with the deepest wells, cf. the concluding remarks at the end of this note.

The asymptotics of the spectral distribution function in the semi-classical limit has applications in quantum mechanics and geometric topology [4–7]. We merely mention Witten’s influential paper [12] and and proofs of the Cheeger–Müller theorem [1–3]. In these geometric applications, a Morse function *f* provides a deformation of the deRham differential, $d_t=e^{-tf}de^{tf}=d+tdf$. The corresponding Witten Laplacian, $\Delta _t=(d_t+d_t^*)^2=d_td_t^*+d_t^*d_t=[d_t,d_t^*]$, is a one parameter family of operators acting on differential forms, i.e., $E=\Lambda ^*T^*M$, which is of the type considered here with scalar valued $V=|df|^2$. Hence, in this case the absolute minima of *V* coincide with the critical points of *f*.

Let us return to a general one parameter family of operators considered above, see (1), and an analytic eigenbranch as in (2). Subsequently, we will use the notation $\dot{\lambda }_t:=\frac{\partial }{\partial t}\lambda _t$, $\dot{\psi }_t:=\frac{\partial }{\partial t}\psi _t$, and

3 $$\begin{aligned} \dot{\Delta }_t:=\tfrac{\partial }{\partial t}\Delta _t=A+2tV. \end{aligned}$$

We have the following analogue of Theorem 1 in [8].

## Theorem

For each analytic eigenbranch the following hold true:

- $$\dot{\lambda }_t=O(t)$$ and $$\lambda _t=O(t^2)$$, as $$t\rightarrow \infty $$.
- $t^{-2}\lambda _t$ converges to a finite limit,
  4 $$\begin{aligned} \mu :=\lim _{t\rightarrow \infty }t^{-2}\lambda _t. \end{aligned}$$
- $t\frac{\partial }{\partial t}(t^{-2}\lambda _t)$ is bounded and
  $$\begin{aligned} \limsup _{t\rightarrow \infty }t\tfrac{\partial }{\partial t}(t^{-2}\lambda _t)=0. \end{aligned}$$
- $t^{-2}\langle \!\langle \Delta \psi _t,\psi _t\rangle \!\rangle$ is bounded and
  $$\begin{aligned} \liminf _{t\rightarrow \infty }t^{-2}\langle \!\langle \Delta \psi _t,\psi _t\rangle \!\rangle =0. \end{aligned}$$
- The limit, $\mu$, has the following interpretations:
  $$\begin{aligned} \mu =\lim _{t\rightarrow \infty }t^{-2}\lambda _t =\limsup _{t\rightarrow \infty }\langle \!\langle V\psi _t,\psi _t\rangle \!\rangle =\limsup _{t\rightarrow \infty }(2t)^{-1}\dot{\lambda }_t =\limsup _{t\rightarrow \infty }\tfrac{\partial }{\partial t}(t^{-1}\lambda _t). \end{aligned}$$

Furthermore, for each sequence $t_n\rightarrow \infty$ such that, cf. (d),

5 $$\begin{aligned} \lim _{n\rightarrow \infty }t_n^{-2}\langle \!\langle \Delta \psi _{t_n},\psi _{t_n}\rangle \!\rangle =0, \end{aligned}$$

the following hold true:

(f) For every positive integer $s\in \mathbb N$,
  $$\begin{aligned} \lim _{n\rightarrow \infty }t_n^{-s}\Vert \psi _{t_n}\Vert _{H^s(M)}=0, \end{aligned}$$
  where $\Vert {-}\Vert _{H^s(M)}$ denotes any Sobolev *s* norm on $\Gamma (E)$.
(g) We have
  6 $$\begin{aligned} \lim _{n\rightarrow \infty }\Vert (V-\mu )\psi _{t_n}\Vert =0. \end{aligned}$$
  Hence, the eigensections $\psi _{t_n}$ localize near $\Sigma _\mu :=\{x\in M:\det (V(x)-\mu )=0\}$. In particular, for every open neighborhood *U* of $\Sigma _\mu$, we have
  7 $$\begin{aligned} \lim _{n\rightarrow \infty }\Vert \psi _{t_n}\Vert _{L^2(M{\setminus } U)}=0. \end{aligned}$$
(h) If, moreover, the potential *V* is multiplication by a (scalar) function, then
  8 $$\begin{aligned} \lim _{n\rightarrow \infty }\bigl \Vert \, |dV|^2\psi _{t_n}\,\bigr \Vert =0. \end{aligned}$$
  Hence, the eigensections $\psi _{t_n}$ localize near the critical points of *V*. For every neighborhood $\tilde{U}$ of the critical set of *V* we have
  9 $$\begin{aligned} \lim _{n\rightarrow \infty }\Vert \psi _{t_n}\Vert _{L^2(M{\setminus }\tilde{U})}=0. \end{aligned}$$
  In particular, $\mu$ has to be a critical value of *V*.

## Proof

From (2) we obtain

10 $$\begin{aligned} \langle \!\langle \Delta _t\psi _t,\psi _t\rangle \!\rangle =\lambda _t. \end{aligned}$$

Differentiating the second equation in (2) we get

$$\begin{aligned} \langle \!\langle \dot{\psi }_t,\psi _t\rangle \!\rangle +\langle \!\langle \psi _t,\dot{\psi }_t\rangle \!\rangle =0. \end{aligned}$$

Differentiating (10) and using the selfadjointness of $\Delta _t$ this leads to

11 $$\begin{aligned} \langle \!\langle \dot{\Delta }_t\psi _t,\psi _t\rangle \!\rangle =\dot{\lambda }_t. \end{aligned}$$

Combining this with (3) we obtain

12 $$\begin{aligned} \dot{\lambda }_t=\langle \!\langle (A+2tV)\psi _t,\psi _t\rangle \!\rangle =\langle \!\langle A\psi _t,\psi _t\rangle \!\rangle +2t\langle \!\langle V\psi _t,\psi _t\rangle \!\rangle . \end{aligned}$$

Since *A* and *V* are bounded operators, this implies $\dot{\lambda }_t=O(t)$, whence (a).

From (1) and (3) we immediately get

$$\begin{aligned} 2\Delta _t-t\dot{\Delta }_t=2\Delta +tA. \end{aligned}$$

Combining this with (10) and (11) and using the boundedness of *A*, we obtain

13 $$\begin{aligned}&\tfrac{\partial }{\partial t}(t^{-2}\lambda _t) =-\,t^{-3}\bigl (2\lambda _t-t\dot{\lambda }_t\bigr ) =-\,t^{-3}\langle \!\langle (2\Delta _t-t\dot{\Delta }_t)\psi _t,\psi _t\rangle \!\rangle \nonumber \\&\quad =-\,t^{-3}\langle \!\langle (2\Delta +tA)\psi _t,\psi _t\rangle \!\rangle =-\,2t^{-3}\langle \!\langle \Delta \psi _t,\psi _t\rangle \!\rangle +O(t^{-2}). \end{aligned}$$

Hence, as $\Delta$ is bounded from below, there exists a constant *C* such that

14 $$\begin{aligned} \tfrac{\partial }{\partial t}\bigl (t^{-2}\lambda _t+Ct^{-1}\bigr )\le 0, \end{aligned}$$

for sufficiently large *t*. This shows that the quantity $t^{-2}\lambda _t+Ct^{-1}$ is monotone, for large *t*. In view of (a) it is bounded too. Whence $t^{-2}\lambda _t+Ct^{-1}$ converges, as $t\rightarrow \infty$. This immediately implies (b).

Similarly, one can show (c) and (d): Rewriting (13) we get

15 $$\begin{aligned} -t\tfrac{\partial }{\partial t}(t^{-2}\lambda _t) =2t^{-2}\langle \!\langle \Delta \psi _t,\psi _t\rangle \!\rangle +O(t^{-1}). \end{aligned}$$

As $t^{-2}\lambda _t$ is bounded, we must have

16 $$\begin{aligned} \limsup _{t\rightarrow \infty }t\tfrac{\partial }{\partial t}(t^{-2}\lambda _t)\ge 0, \end{aligned}$$

for otherwise $t^{-2}\lambda _t$ would diverge logarithmically. Moreover,

17 $$\begin{aligned} \liminf _{t\rightarrow \infty }t^{-2}\langle \!\langle \Delta \psi _t,\psi _t\rangle \!\rangle \ge 0, \end{aligned}$$

since $\Delta$ is bounded from below. Combining (15)–(17) we obtain

$$\begin{aligned} \limsup _{t\rightarrow \infty }t\tfrac{\partial }{\partial t}(t^{-2}\lambda _t)=0=\liminf _{t\rightarrow \infty }t^{-2}\langle \!\langle \Delta \psi _t,\psi _t\rangle \!\rangle . \end{aligned}$$

This completes the proof of (c) and (d), the statements on boundedness follow immediately from (a) and (15).

To see (e) note that (1) and (10) give

$$\begin{aligned} t^{-2}\lambda _t=t^{-2}\langle \!\langle \Delta \psi _t,\psi _t\rangle \!\rangle +t^{-1}\langle \!\langle A\psi _t,\psi _t\rangle \!\rangle +\langle \!\langle V\psi _t,\psi _t\rangle \!\rangle . \end{aligned}$$

Using (d) we obtain the second equality in (e). The third equality follows from (12). The last one is immediate using $\frac{\partial }{\partial t}(t^{-1}\lambda _t)=t^{-1}\dot{\lambda }_t-t^{-2}\lambda _t$.

To see (f), note first that the case $s=1$ is immediate from (5) since there exists a constant *C* such that $\Vert \psi \Vert _{H^1(M)}^2\le C(\langle \!\langle \Delta \psi ,\psi \rangle \!\rangle +\Vert \psi \Vert ^2)$. Using the eigensection equation $\Delta _t\psi _t=\lambda _t\psi _t$ and (a), one obtains constants $C_s$ such that

$$\begin{aligned} \Vert \psi _t\Vert _{H^{s+2}(M)}\le C_s(1+t^2)\Vert \psi _t\Vert _{H^s(M)}. \end{aligned}$$

This permits to establish (f) inductively for all odd integers $s\in \mathbb N$. The even case can be reduced to the odd one using $\Vert \psi \Vert ^2_{H^2(M)}\le C'\Vert \psi \Vert _{H^1(M)}\Vert \psi \Vert _{H^3(M)}$, an estimate which readily follows from the Cauchy–Schwarz inequality.

Using the estimate in (f) for $s=2$, the eigensection equation,

$$\begin{aligned} t^{-2}\Delta \psi _t+t^{-1}A\psi _t+(V-\mu )\psi _t=(t^{-2}\lambda _t-\mu )\psi _t, \end{aligned}$$

implies (6). As $V-\mu$ is invertible over the compact set $M{\setminus } U$, there exists a constant $c>0$ such that $c^2\le (V-\mu )^*(V-\mu )$, over each point in $M{\setminus } U$. Consequently,

$$\begin{aligned} c\,\Vert \psi \Vert _{L^2(M{\setminus } U)}\le \Vert (V-\mu )\psi \Vert _{L^2(M{\setminus } U)}. \end{aligned}$$

Combining this with (6), we obtain (7).

Let us now turn to the proof of (h). Suppose *D* is a first order differential operator acting on sections of *E*. Since *V* is scalar valued, the commutator $\sigma :=[D,V]=DV-VD$ is a differential operator of order zero, namely the principal symbol, $\sigma =\sigma _D(dV)\in \Gamma ({{\,\mathrm{end}\,}}(E))$. As $\Delta$ is a Laplacian, the commutator $[D,\Delta ]$ is a differential operator of order at most two. Using

$$\begin{aligned} {[}D,t^{-2}\Delta _t]=t^{-2}[D,\Delta ]+t^{-1}[D,A]+\sigma , \end{aligned}$$

the Cauchy–Schwarz inequality and (f), we thus obtain

18 $$\begin{aligned} \Vert \sigma \psi _{t_n}\Vert ^2=\langle \!\langle [D,t_n^{-2}\Delta _{t_n}]\psi _{t_n},\sigma \psi _{t_n}\rangle \!\rangle +o(1),\qquad \text {as }n\rightarrow \infty . \end{aligned}$$

Using $\Delta _t\psi _t=\lambda _t\psi _t$ one readily checks

19 $$\begin{aligned} \langle \!\langle [D,t^{-2}\Delta _t]\psi _t,\sigma \psi _t\rangle \!\rangle =\langle \!\langle D\psi _t,[\sigma ,t^{-2}\Delta _t]\psi _t\rangle \!\rangle . \end{aligned}$$

Since *V* is scalar valued we have $[\sigma ,V]=0$ and

$$\begin{aligned} t[\sigma ,t^{-2}\Delta _t]=t^{-1}[\sigma ,\Delta ]+[\sigma ,A], \end{aligned}$$

where $[\sigma ,\Delta ]$ is a differential operator of order at most one. Proceeding as above, the Cauchy–Schwarz inequality and (f) yield

$$\begin{aligned} \lim _{n\rightarrow \infty }\langle \!\langle D\psi _{t_n},[\sigma ,t_n^{-2}\Delta _{t_n}]\psi _{t_n}\rangle \!\rangle =0. \end{aligned}$$

Combining the latter with (18) and (19), we arrive at

20 $$\begin{aligned} \lim _{n\rightarrow \infty }\Vert \sigma \psi _{t_n}\Vert =0. \end{aligned}$$

Specializing to $D=\nabla _X$ where $\nabla$ is some linear connection on *E* and *X* is a vector field on *M*, we obtain $\sigma =X\cdot V=dV(X)$. Choosing $X={{\,\mathrm{grad}\,}}(V)$ with respect to some auxiliary Riemannian metric on *M*, we have $\sigma =|dV|^2$, and (20) becomes (8). On $M{\setminus }\tilde{U}$ we have $0<\tilde{c}\le |dV|^2$ for some constant $\tilde{c}$, hence

$$\begin{aligned} \tilde{c}\,\Vert \psi \Vert _{L^2(M{\setminus }\tilde{U})}\le \bigl \Vert \,|dV|^2\psi \bigr \Vert _{L^2(M{\setminus }\tilde{U})}, \end{aligned}$$

and thus (8) implies (9). Combining the latter with (g), we see that $\mu$ has to be a critical value of *V*. This completes the proof of the theorem. $\square$

Let us mention that many of the preceding statements remain true for continuously parametrized eigenbranches. In particular, the limit in (4) exists for every continuous eigenbranch $\lambda _t$, and this limit has to be a critical value of *V*, provided *V* is scalar. Since continuous eigenbranches are piecewise analytic, this can readily be derived by observing that the constants implicit in (a) are uniform for all analytic eigenbranches.

## Concluding remarks

At the very end of Section 3 in [8], Hillairet points out that for $M=\mathbb R^1$ and non-degenerate minima, the limit $\mu$ has to be the absolute minimum of *V*. Indeed, in this situation the spectrum is known to be simple, hence the analytic eigenbranches cannot cross and remain in the same order: $\lambda _t^{(1)}<\lambda _t^{(2)}<\cdots$ The semi-classical asymptotics of the *k*-th eigenvalue, however, is governed by the deepest wells.

For the operators considered in the theorem above we propose the following

### Conjecture

If the minima of *V* are non-degenerate in the sense of Shubin [10, Condition C on p. 378] and *M* is connected, then $t^{-2}\lambda _t$ converges to the absolute minimum of *V*.

From (14) we see that there exists a constant *C* such that

$$\begin{aligned} \mu -Ct^{-1}\le t^{-2}\lambda _t \end{aligned}$$

for sufficiently large *t*. It is unclear to the author, if a similar estimate from above holds true, i.e., if we have $t^{-2}\lambda _t=\mu +O(t^{-1})$ as $t\rightarrow \infty$.

As $t\rightarrow \infty$, the following statements are equivalent:

- $$\langle \!\langle \Delta \psi _t,\psi _t\rangle \!\rangle =O(t)$$
- $$\langle \!\langle (V-\mu )\psi _t,\psi _t\rangle \!\rangle =O(t^{-1})$$
- $\frac{\partial }{\partial t}(t^{-\alpha }(\lambda _t-\mu t^2))=O(t^{-\alpha })$ for one (and then all) real $\alpha \ne 1$.
- $$\frac{\partial }{\partial t}(t^{-2}\lambda _t)=O(t^{-2})$$
- $$\dot{\lambda }_t-2t\mu =O(1)$$

Indeed, the equivalence $\mathrm{(a)}\Leftrightarrow \mathrm{(d)}$ follows from (15), the equivalence $\mathrm{(b)}\Leftrightarrow \mathrm{(e)}$ follows from (12), and the equivalence $\mathrm{(c)}\Leftrightarrow \mathrm{(d)} \Leftrightarrow \mathrm{(e)}$ follows from (4). Moreover, if these five (equivalent) statements hold true, then clearly

21 $$\begin{aligned} t^{-2}\lambda _t=\mu +O(t^{-1}),\qquad \text {as }t\rightarrow \infty . \end{aligned}$$

Suppose (21) holds true. Moreover, assume that $\mu$ is the absolute minimum of *V* and the minima are all non-degenerate in the sense of [10, Condition C on p. 378]. Then, according to [10, Theorem 1.1] or [4, Theorem 11.1], there exists an eigenvalue $\omega$ of the harmonic oscillator associated with the minima (deepest wells) such that

22 $$\begin{aligned} \lambda _t=\mu t^2+\omega t+O(t^{4/5}),\qquad \text {as }t\rightarrow \infty . \end{aligned}$$

Better estimates are available if the geometry is flat near the minima, cf. [10, Equation (1.15)]. Under additional assumptions [11] one even obtains a full asymptotic expansion in terms of integral powers of *t*.

For every eigenvalue $\omega$ of the harmonic oscillator associated with the minima of *V*, there exists an analytic eigenbranch for which (22) holds true with $\mu$ the absolute minimum of *V*. Moreover, the number of these eigenbranches coincides with the multiplicity of $\omega$. However, it is unclear, if these exhaust all analytic eigenbranches. Hence, an intriguing problem remains open: Are there analytic eigenbranches with different asymptotics which are not governed by the approximating harmonic oscillator associated with the deepest wells?
